# Biocompatibility of Polyimides: A Mini-Review

**DOI:** 10.3390/ma12193166

**Published:** 2019-09-27

**Authors:** Catalin P. Constantin, Magdalena Aflori, Radu F. Damian, Radu D. Rusu

**Affiliations:** 1 “Petru Poni” Institute of Macromolecular Chemistry, Romanian Academy, Aleea Grigore Ghica Voda 41A, Iasi-700487, Romania; constantin.catalin@icmpp.ro (C.P.C.); maflori@icmpp.ro (M.A.); 2 SC Intelectro Iasi SRL, Str. Iancu Bacalu, nr.3, Iasi-700029, Romania; rdamian@etti.tuiasi.ro

**Keywords:** biocompatibility, polyimides, polyimide films, biostability, structural biocompatibility, in vitro testing, in vivo screening

## Abstract

Polyimides (PIs) represent a benchmark for high-performance polymers on the basis of a remarkable collection of valuable traits and accessible production pathways and therefore have incited serious attention from the ever-demanding medical field. Their characteristics make them suitable for service in hostile environments and purification or sterilization by robust methods, as requested by most biomedical applications. Even if PIs are generally regarded as “biocompatible”, proper analysis and understanding of their biocompatibility and safe use in biological systems deeply needed. This mini-review is designed to encompass some of the most robust available research on the biocompatibility of various commercial or noncommercial PIs and to comprehend their potential in the biomedical area. Therefore, it considers (i) the newest concepts in the field, (ii) the chemical, (iii) physical, or (iv) manufacturing elements of PIs that could affect the subsequent biocompatibility, and, last but not least, (v) in vitro and in vivo biocompatibility assessment and (vi) reachable clinical trials involving defined polyimide structures. The main conclusion is that various PIs have the capacity to accommodate in vivo conditions in which they are able to function for a long time and can be judiciously certified as biocompatible.

## 1. Introduction

Six decades after their entry into the market of commercial polymeric materials for aerospace, defense, and optoelectronics, polyimides (PIs) still maintain their status as a benchmark for high-performance polymers on the basis of a remarkable collection of thermoxidative resistance, outstanding mechanical characteristics, long-term reliance, and accessible production pathways [1,2,3,4,5,6,7]. The reputation of this engineering polymer family among advanced-technology industries is backed by a handful of traits: excellent dielectric features, strength and flexibility, chemical and radiation inertness, interesting optical attributes, durability, prolonged shelf life, and others [8,9,10]. As a result, PIs easily established a plethora of applications as (thermoplastic or thermosetting) dielectric films, wire insulation coatings, advanced composite matrices, specialty adhesives, and molding resins, photoimageable layers, flexible circuits, non-woven materials, advanced fabrics, medical tubing, etc. [7,8,9,10]. As expected, all these commercially-relevant features come with a premium cost. Nevertheless, the certified versatility and performance of this polymeric family render PIs as the materials of choice for high-performance, advanced uses in which breakdown, malfunction, or replacement costs are even higher [2,7,11,12].

Due to this remarkable combination of features, PIs incited serious attention from the exponentially growing medical field. This was doubled by the already accumulated knowledge regarding their functionalization, tuning, and processing which can be used to satisfy manifold complex requirements of present biomedical topics [12]. Therefore, several polyimide-based materials have been explored in the last two and a half decades in various biomedical applications, from micropatterned cell substrates to implantable neuronal sensors. The main drive of research in this area is mainly focused on novel, processable, tailorable PI materials of superior performance to satisfy the innovation of the future. However, special interest must be directed towards their ability to make it in the ever-demanding biomedical field, biocompatibility being the first item on a long checklist.

Biocompatibility represents a complex endeavor for producers of biomaterials and medical devices. It is influenced by the entire lifecycle of a product, from the initial stages of design and materials development up to the market clearance and entry, and is the subject of tight regulations, guidance, and supervision. Hence, biocompatibility assessment embodies a composite enterprise of many variables, from the chemical nature or physical characteristics of the envisaged material to the tissue contact span or the nature of that tissue. Therefore, some pointers and perspectives are necessary when a (an already intricate and expensive to manufacture, high value) material arrives at the point of selecting the proper (also elaborate and high-priced) in vitro or in vivo biocompatibility evaluation technique [13,14].

PIs are sometimes described as “biocompatible” based on the seminal work presented in [15], which is dated 1993 and deals with the in vitro evaluation of five commercial polyimidic products. Even so, up-to-date, proper analysis of their biocompatibility and safe use in biological systems is deeply needed. Many times, a PI-based material designed for, e.g., further implanting is reported as biocompatible only by (in the best case scenario) citing the above-mentioned research or on the basis of some scarcely described in vitro cytotoxicity tests. On other occasions, their reputation can be at risk due to research performed on generally-described “polyimide-based” biomedical devices with poor results [16]. PIs are engineered starting from a rather small collection of building blocks which nevertheless provide a broad range of PI formulations. This wide PI variety demands more data, interpretations, and correlations to achieve the “biocompatible” label since even a small change in the macromolecular architecture can generate noticeable differences in terms of cytotoxicity, thrombogenicity, interaction with various cells and tissues, biostability and many others [17,18]. Ultimately, each polyimidic material intended to be employed in any biomedical application needs to be severely tested. However, a starting point on the topic is imperative and, in the absence of any solid known review dedicated to the matter, the present work aims to take the first steps in this regard.

This mini-review is designed to encompass some of the most robust available research on the biocompatibility of various commercial or in-house synthesized PIs and to comprehend their potential in the biomedical area. It follows all reachable articles related to the biocompatibility of polyimides published in the last two decades, as present in the Web of Science database (topic: “biocompatibility” and “polyimide”, timespan: 2000–2019, all databases). The research was performed by considering (i) the newest concepts in the field, (ii) the chemical, (iii) physical, or (iv) manufacturing elements of PIs that could affect the subsequent biocompatibility, and, last but not least, (v) in vitro and in vivo biocompatibility assessment and (vi) reachable clinical trials involving defined polyimide structures.

## 2. Polyimides: Synthesis, General Features, and Biomedical Applications

PIs represent a family of polymeric architectures acknowledged first and foremost for their high-temperature resistance, which covers all their material forms: films, coatings, varnishes, binders, tapes, fibers, composites, and foams. The chemistry of PIs is by nature a broad domain of materials science and covers a wide assortment of macromolecular backbones, starting materials, and synthetic pathways. Therefore, they are able to deliver manifold characteristics covering quite some large boundaries, which can be easily amended by relatively inconsequential structural modifications. There are many excellent monographs, reviews, and books dedicated to this class of polymers and the present work does not intend to reproduce their content, but to resume the essentials and to build the basics for the upcoming chapters [1,2,3,7,8,11]. This section is dedicated to aromatic polyimides, which are planar imidic macromolecules containing rigid aromatic rings and various flexible, heteroaliphatic, unsymmetrical, bulky or pendant structural motifs. We chose to focus on the aromatic polymers since the majority of polyimidic materials tested for biomedical uses subscribe to this structural architecture, even if some commercial or in-house aliphatic backbones have found their way into the field [19,20]. Moreover, the review is mainly centered on polyimidic films (also encompassing foils and coatings) since, with some noticeable exceptions which will be underlined at their rightful place, this is the format chosen by most bio-related applications and devices.

**Scheme 1:**
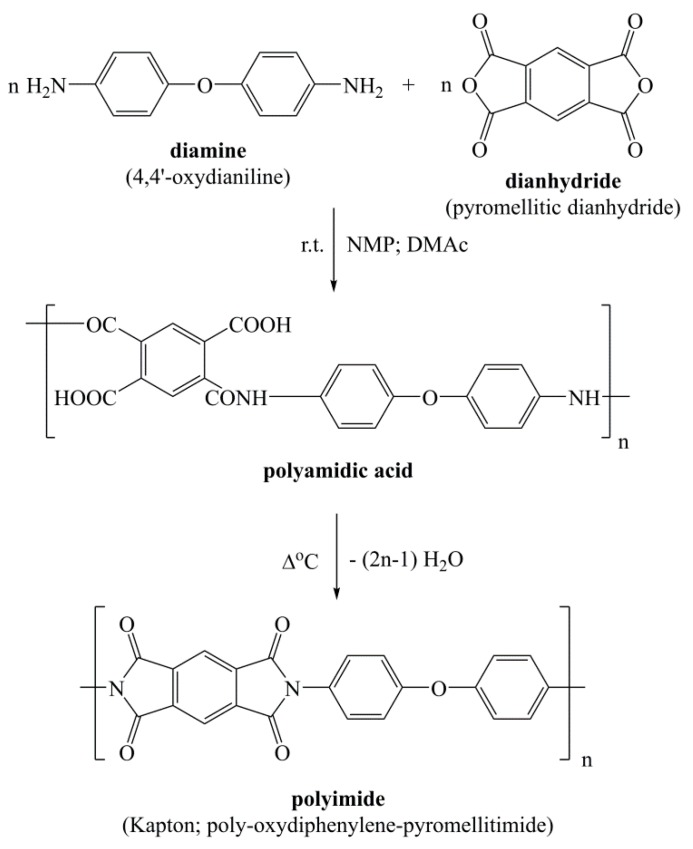
Schematic representation of the two-step synthesis of a conventional polyimide.

In general terms, polyimides are attained by a one- or two-stage reaction between a diamine and a dianhydride in a polar aprotic solvent (N,N-dimethylacetamide and N-methyl pyrrolidinone being the most used ones) which follows a polycondensation mechanism (although a polyaddition process is also available). The two-stage pathway (Scheme 1) implies the formation of a highly soluble, intermediate polyamic acid (or polyamidic acid, PAA) through a mildly exothermic reaction between the starting compounds at modest temperatures (room temperature usually suffices). This is a stepwise process that takes place at a rate depending on the monomers’ reactivity. Afterward, the PAA intermediate is transformed into the corresponding polyimidic structure via thermal or chemical imidization and concurrent H_2_O release. This procedure is usually employed in laboratories or by industry in the preparation of polyimidic materials which are rather insoluble in their final form [2,21,22].

The one-stage pathway eliminates the PAA precursor from the process and involves the stepwise polymerization of the (at least) two monomers at high temperatures and concomitant thermal (sometimes chemical) imidization of the macromolecular backbone. This procedure is mainly followed in-house in the synthesis of soluble, readily processable polyimides [1,23,24].

The PAA route is the most used technique to produce commercial PI films. Usually, the PAA precursor is stable and displays a long shelf life, which makes it easy to handle, deposit, transport, or shape it into films, coatings, or fibers. PAA films are cast or spin-coated, and afterward thermally dehydrated to generate the final imide film. The main drawback of this process is inefficient imidization (cyclization) along the polymer chain, which usually does not render major flaws in the final material. The imidization reaction implies only the release of water, which can be easily removed nowadays by both small- or large-scale manufacturing, and the obtained polyimidic films, foils or coatings are mostly free of voids or defects.

Based on these simple and straightforward synthetic methods, most PIs can be relatively amended and tailored to supply various features. This is usually performed based on judicious monomer modifications through proper chemical tools or, to a less extent, by small interventions within the manufacturing process, with little sacrifice of the desired properties of the final material. The first approach is used on a large scale to resolve PIs’ biggest bottleneck: reduced solubility, high softening temperatures, and troublesome or costly processing coming from highly symmetrical, polar, and rigid macromolecular backbones with strong intra- and interchain interactions.

The same structural elements mentioned above unlock the high-performance character of PIs: thermoxidative stamina, chemical (organic solvents included) and mechanical durability, excellent insulating properties, and long-term reliance. These traits make them suitable for service in hostile environments and purification/sterilization by robust methods, as requested by most biomedical applications. Moreover, as it will be detailed later, PIs have the capacity to accommodate to in vivo conditions in which they are able to function for a long time without a harming response.

**Figure 1:**
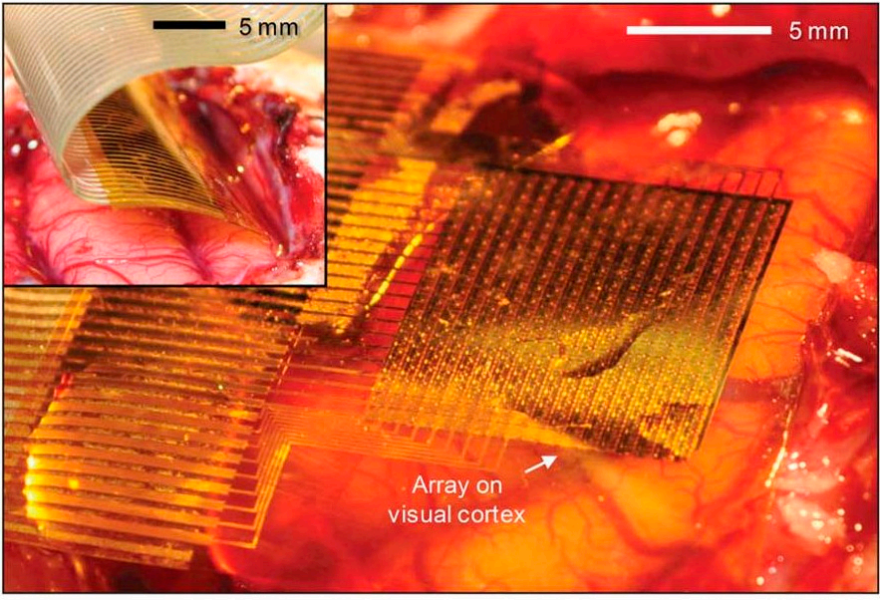
A high-density array of electrodes for mapping neural activity: flexible electrode array insulated and encapsulated by polyimide (unknown trademark) layers placed on the visual cortex of a cat; (inset) the folded electrode array inserted into the interhemispheric fissure. Adapted from [26], with permission from © 2011 Springer Nature.

All of the above and the positive experience of heavily employing PIs in microelectronics lead to the successful use of polyimidic materials in high end, complex biomedical devices like bio micro-electromechanical-systems (bio-MEMS), e.g., retinal and neural implants [16,25]. PIs fulfill a three-fold function in these devices: a flexible (and unstretchable) mechanic substrate for the embedded metallic elements, an efficient insulating substrate between adjacent conductor paths and an overall (chemical) encapsulant to protect the device against the adverse liquid surroundings. Depending on the final application, PIs enable the production of bio-MEMS devices of small- or micro-sizes and thickness, various shapes, high density of electrodes, a large number of stimulating/recording elements, and variable degrees of flexibility or stiffness. As a consequence, the use of PIs ensures the smallest possible intrusion in the biological frame, an additional key feature for implantable devices. Moreover, PIs are already easily processed by well-established micromachining techniques in conventional clean room facilities which are also employed in the production of bio-MEMS, thus ensuring the desired repeatability, pattern accuracy, and low costs [7,8,16]. The complexity of such a device is depicted in Figure 1, showing a flexible, stacked, polymer-metal-polymer microsystem placed on the visual cortex of a cat.

In a broad sense, there are two main PI versions employed in biomedical research: conventional, non-photosensitive PI materials (cPIs), and photosensitive polyimides (psPIs). The latter contain photoreactive side- or end-groups, usually of acrylic nature. Representatives of both versions are commercially available mostly as aromatic polymers. cPIs are typically used starting from their PAA precursors, while psPIs are mostly based on PAA variations, namely esters or salts of their polyamic acids [27,28]. Both forms are available as liquids or varnishes, which are cast or spin-coated on various substrates and thermally cured until an imidized film is obtained. Some commercial cPIs are directly available and employed as preimidized films, like the well-known Kapton or Ultem polyimidic materials (for the actual structures of the most used commercial PIs see Table 1). cPI films are processed by photolithography and dry/wet etching which imply a photoresist or hard mask as an etch template. The psPI counterparts are manufactured by straightforward patterning through UV exposure and developer chemicals, which eliminate some processing steps. As a drawback, the photo-definable versions are moisture sensitive and prone to water sorption and uptake, which needs additional stabilizers or restricts their application in vivo [25].

The properties of each commercial polyimidic film can fluctuate broadly even within the same PI version (cPI or psPI), due to structural features, manufacturing technique, imidization temperature, and final dimensions of the material, which can inflict modifications in terms of density, polymer chain orientation, mechanical features, and moisture uptake. The issue becomes even more complicated when it comes to the evaluation of PI materials of noncommercial nature (synthesized in the laboratory). The plethora of aliphatic, semialiphatic, or aromatic PI structures makes it simply impossible to include their properties into clearly defined boundaries. Fortunately or not, the number of PIs which were tested for their biocompatibility is relatively small, some of their structures being presented in Table 2.

## 3. Biocompatibility: Concepts, Standards, and Assessment

The term “biocompatibility”, the “holy grail” of any researcher working in the biomaterial or medical domains, has reached such a common and careless usage in countless areas and contexts that its sense and gravity have been attenuated and blurred [17]. On one side, there is a constant fluctuation in its operation and significance. On the other, the modern exponential evolution of biology and medicine generates several mutations in the concepts and understanding of biocompatibility. The present section is designed to touch some key notions and attributes related to the field and place them in a contemporary context.

In its beginnings, biocompatibility was regarded as the property of a material to be neutral from the physical, chemical, and physiological point of view. Nowadays, the term has progressed to an intricate concept describing the interaction between a material and a living biological framework. The definition accepted by the academic majority is “the ability of a material to perform with an appropriate host response in a specific application”. There is also an expanded version that includes the concept of “bioactivity” (an action stimulated by the material) and thus becomes much wider than simple inertia [17,18]. This comes as a necessary complement of the IUPAC (International Union of Pure and Applied Chemistry) definition which describes biocompatibility as the “ability to be in contact with a living system without producing an adverse effect” [56]. For a detailed and intriguing discussion regarding the meaning of “biocompatibility” and its often improper uses, the reader is directed towards reference [13], in which is judiciously argued that “a biocompatible material“ is an abuse and the proper descriptive expression is “intrinsically biocompatible system”.

Biocompatibility assessments pursue the potential risk brought by a material for a living organism, e.g., patient, by employing certain conditions and environments which are close or similar to clinical circumstances. Naturally, the multiplicity which governs biomaterials, their applications and interactions with biological systems makes it difficult or even impossible to provide a harmonized, generally accepted, or practical evaluation of biocompatibility [13,14,17]. The assessment of (implantable) medical devices, the main bio-related application of polyimidic materials, is established by internationally acknowledged standards. The ISO-10993 (International Organization for Standardization) guidelines are the most important and used standards in Europe, Asia (most countries) and the US (even if the FDA (U.S. Food and Drug Administration) provides some more severe requisites on certain topics). These guidelines are not to be considered as a mandatory checklist; they build a recommended general framework of suitable, efficient tests for the analysis of biocompatibility, and judicious proofs of equivalence are accepted [18].

At this point, we must underline that, currently, there is no PI-based material certified according to the above-mentioned standards. According to our assessment, the BPDA-PPD polyimidic architecture (abbreviated accordingly to the starting monomers: diphenyl dianhydride, BPDA, and *p*-phenilene diamine, PPD) is the most used polyimidic material in bio-related studies and applications, being the main subject of roughly 24% of the articles analyzed for this review. Even this material, commercially available as PI2611 (DuPont; HD Microsystems) or U-Varnish-S (UBE), fails in this regard. Nevertheless, several research teams explored its application in neural interfaces and demonstrated its overall biocompatibility as a bulk material or encapsulant of a long-term implantable device, based on in vitro and in vivo assays, as it will be discussed later [16,25].

**Figure 2:**
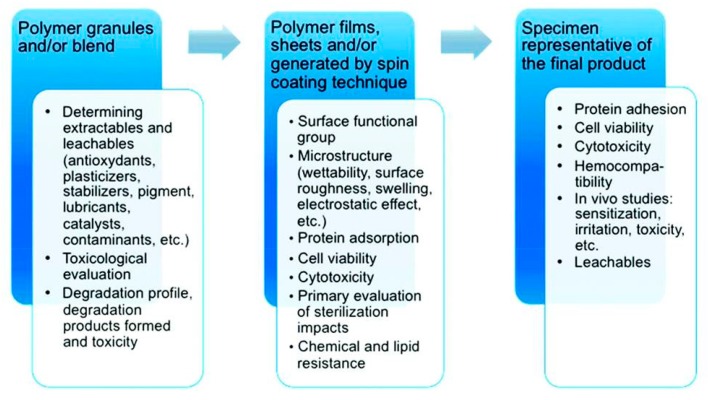
Sequential chemical, physical, and biological investigation steps of polymeric materials for biocompatibility assessment. Adapted from [18], with permission from © 2018 Royal Society of Chemistry.

Biocompatibility tests can be partitioned on three levels: in vitro (effects of materials on cultured cells), in vivo (tissue reactions after implantation, usually in animals), and preclinical tests (performance and reactions of biomaterials in normal clinical conditions after implantation in humans) [57]. They are assessing “the big three” biological effects: cytotoxicity (in vitro), sensitization (in vivo), and irritation (in vivo), which are common to biomaterials designed for medical devices. Despite the absence of the complex biological and physiological environment, in vitro studies are employed on a large scale and are able to provide valuable information regarding the biological toxicity of a given material and to direct further studies (Figure 2). They involve lower costs and, most important, a smaller extent of ethical controversies as compared to in vivo experiments, which are nevertheless closer to an ideal biological research methodology [14,18,57]. Both types of screening imply a robust research methodology and justification which can be attained by a thorough, sequential chemical and physical investigation.

Therefore, biocompatibility reviewing is a complex process that follows a gradual investigative system that is influenced by several parameters coming from the material. In the particular case of polymers, polyimides included, these parameters are bulk characteristics (overall composition, degree of crystallinity, morphology), surface features (surface chemistry and energy, hydrophilicity, morphology), degradation kinetics and products, leachables and extractables and their potential toxicity. An ideal pattern for comprehending and assessing all these features does not exist, but the systematic approach provided in [17,18] is a good starting point. However, the employment of standardized analyses should become the generally accepted rule, since (especially in a scientific environment inclined towards open access) it enables judicious comparisons between various studies and empowers knowledge related to the biological behavior of materials and their biosafety.

### Biostability of Polyimides

When it comes to biomaterials designed for implantable medical devices, biostability is a concept which needs a separate discussion. This is also the case of PIs since most of their bio-related applications deal with long-term implantable devices, e.g., neural prostheses. In a broad sense, biostability implies various aspects, mainly of chemical and sometimes mechanical nature, related to the stability and integrity of the investigated polymeric material or the system containing it. For example, the mechanical support and electrical or chemical insulating layer prepared from one or several PI films should not degrade or delaminate in the harsh environment provided by the human body. The good adhesion between metals and PIs is certified by their long common use in microelectronics. However, the delamination of metals (Ti, Au, Pt) from PIs is a serious issue for the long-term durability of bio-MEMS and a threat for their host [16,25].

The aqueous surroundings of an implant presume interactions between the PI material and different ions and molecules of various sizes (salts and proteins) and a certain amount of absorbed water. While the former does not pose a threat due to the general chemical inertness of PIs, water uptake can be a two-fold problem even for these generally hydrophobic polymers. Moisture adsorption causes stretching and has a plasticizing effect, which becomes a serious issue for the long-term use of PIs, especially for the more moisture-sensitive psPI type. Moreover, any hydrolytic-induced alteration or degradation can be exacerbated by pH changes of the environment and, in the case of bio-MEMS, by voltages coming from the microelectrode arrays. A common and accessible approximation of a polymeric material’s biostability can be performed by in vitro soaking tests in various media (saline physiologic solutions, saline buffer salts, cell culture media, etc.) at body temperature [25]. Sometimes, these biostability assays are accomplished in accelerated aging conditions (at higher temperatures in the same media) in order to boost diffusion and moisture uptake and asses the influence of water and aging and to estimate a failure period.

One of the least polar PIs, BPDA-PPD, excels in this regard since it displays a very low water uptake (0.045%) and a subsequently reduced coefficient of expansion induced by the wet environment [16,29,44]. This is one of the main reasons for the extensive study of its commercial forms (liquid varnish precursors PI2611 and U-Varnish-S, thermally cured in-house until imidization) as the polymeric support and encapsulant of many neuroprosthetics. This polymeric material raises above other frequently employed PIs, like Kapton, several psPIs, fluorinated PIs, and other BPDA-based PIs, which have a much higher water uptake or, in some extreme cases, prove slow degradation in the wet environment [1,11,16,25,58]. Both BPDA-PPD-based commercial PIs are quite stable to the plasticization effect of water. More importantly, they are able to sustain excellent mechanical features after long-term storage in simulated body conditions (20 months at 37 °C in water or saline phosphate buffer, PBS) or after accelerated aging (60 and 85 °C in water) [29]. These observations are supported by several studies regarding the in vivo assessment of electrodes based on the aforementioned PIs, which showed them to properly operate even at 12 months post-implantation [59,60].

Subjected to the same simulated body conditions as PI2611 and U-Varnish-S, a commercial psPI material (Durimide 7510; Fujifilm) also showed surprising long-term stability in saline buffer and deionized water environments, despite a small, yet tolerable decrease of the mechanical characteristics [29]. None of the three investigated PIs showed any visible changes in the chemical composition of the macromolecular backbone, as evaluated by infrared spectroscopy.

However, all of them displayed a decrease of the mechanical characteristics after being stored for a long period (20 months) in PBS at 85 °C, indicating an undeniable sensitivity to alkali metal salts and ions at this temperature.

**Figure 3:**
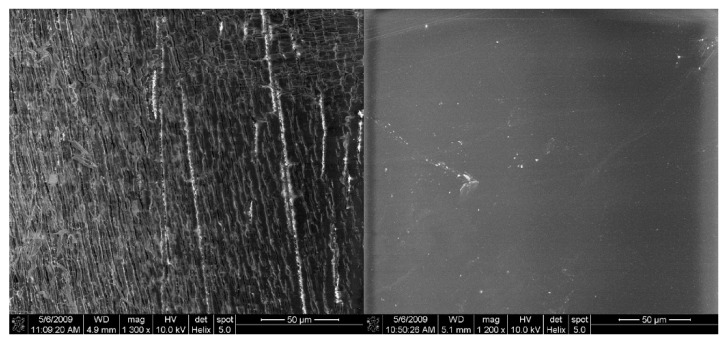
SEM micrographs of the surface of Upilex25S film after (left) and before (right) being incubated in PBS at 85 °C for 18 months. Reproduced from [29], with permission from © 2010 Elsevier.

The Upilex 25S foil, a commercial preimidized version of BPDA-PPD liquid precursors, provided resembling stability at body temperature in water and PBS. When exposed to more extreme experimental conditions, it showed a mass loss and some mutations in the overall macroscopic behavior. For example, after being incubated in PBS at 85 °C for 18 months, this PI foil displayed a heterogeneous area with changed rugosity and revealed several creases on its surface (Figure 3). Moreover, it started to tear apart while carefully being handled or rinsing. All these observations corroborate to the reasoning that at least some parts of the materials, most likely uncyclized polyamic acid, dissolved in PBS at high temperatures.

Another conclusion coming from these studies is that proper attention must be directed towards the experimental conditions in which accelerated aging assays are performed. The mechanisms by which polyimidic materials are failing in vitro are not always dependent on the diffusion or uptake of water and temperature-subordinated processes must be taken into consideration, alone or in combination with the former. For example, the aforementioned incubation in PBS at 85 °C for various months does not prove to be a proper method to evaluate the long-term conduct of PIs at body temperature.

Moreover, any of these results require validation by in vivo testing, and the ISO standards provide some guidance for finding the right experiments in this regard. These evaluations also need to approach any structural changes taking place in the material after implantation. For instance, one brief paper on the topic reports delamination and a 30% drop in the tensile strength of PI2611 after 11 months in vivo [61]. This difference underlines that in vitro models and experiments are not able to encompass the potential effect of enzymes on the PI material. Therefore, even if PBS is much closer to the body environment as compared to water in terms of salt or ions content and pH, in vitro soaking tests in this medium can give results quite far from the in vivo ones. At first glance, it seems futile to particularize the differences between in vitro and in vivo assays, even if strictly referring to polyimidic materials. Nonetheless, there are several studies which try to draw a direct line between the two, an at least risky, if not erroneous endeavor.

Taking into consideration all of the above, it seems rational to accede to the necessity of a paradigm change regarding implants proposed in [17], and replace “biocompatibility” with “biotolerability”, which is defined as “the ability of materials to reside in the body for long periods of time with only low degrees of inflammatory reaction”.

## 4. The Influence of Chemical Composition, Physical Features, and Manufacturing of Polyimides on Their Biocompatibility Outcome

Despite the intensive research in the field, the basic elements which control polymers’ biocompatibility are not yet fully comprehended. This is especially the case of the chemical building blocks or the overall chemical blueprint that govern the interaction of an implanted polymeric biomaterial with the host system. This is available for polyimides as well and a straight line between the chemical formulation of a polyimidic material and its biocompatibility is inaccessible. On the other side, any correlation between important factors like interfacial free energy or molecular weight and biocompatibility is unreachable due to disparate or missing information in the available literature. However, based on several biocompatibility assays related to polyimides, we identified some key points which connect the chemical structure or surface of polyimides and the biocompatibility outcome of the resulting materials. These are addressed in the following subsections and are related to (i) the chemical nature of the secondary components of the polyimidic material (and a particular case related to functional groups’ influence), (ii) the surface wettability (balance between the hydrophilicity and the hydrophobicity), (iii) mechanical features of the polymeric film, (iv) surface topography or roughness.

### 4.1. Chemical Composition

The assessment of biocompatibility also covers the evaluation of any biochemical reaction determined by the interaction between the biological framework and the overall material or its secondary components. The latter are generally called leachables or extractables and display a variety of sources: processing aids, unremoved solvents, antioxidants, stabilizers, plasticizers, catalysts, oligomers, monomers, contaminants, degradation products or debris, etc. [17,18]. As detailed earlier, PI films and foils come from straightforward synthetic and manufacturing pathways and display overall chemical resistance and tolerance to conventional sterilization (with a soft spot for alkali or aqueous solutions). These features drastically reduce the long list of leachable sources and increase the importance of sample preparation and investigation in the overall biocompatibility outcome According to the ISO guidelines, an extraction ratio (sample surface area/extractant volume) of 6 cm^2^/mL is recommended for polymeric films with a thickness below 0.5 mm. The extraction media needs to properly represent the environment in which the material or device is intended for use. Likewise, the extraction conditions must come close to the circumstances in which the material is going to be used. However, in many cases, the available studies on polyimides’ biocompatibility deviate from these guidelines and a comparison between their outcomes must be done with caution. Of course, these guidelines should be applied only in the case of properly purified polyimidic materials, an attribute which, unfortunately, is not found in all scientific papers dedicated to PIs.

There are many factors that can lead to a large variation in the main features of PI materials and affect their biocompatibility and their post-implant behavior. Alongside structural diversity, commercial availability of two forms of the same material (PAA precursor and preimidized film), small variations in the processing methods (different curing temperatures included) or even different sizes of the same sample are some the most common reasons for different biocompatibility outcomes of polyimidic materials. For example, the relatively high water uptake and moisture sensitivity of Kapton, perhaps the most known and generally used PI, hampers its long-term application in bio-MEMS. In comparison, the PI2611 precursor or its Upilex 25S cyclized counterpart do not suffer from such a disadvantage [29]. As detailed in the previous section, the PI’s stability reaches its maximum in the fully cured state, that is when the PAA amount is reduced to a minimum by its proper treatment at elevated temperatures [27].

**Figure 4:**
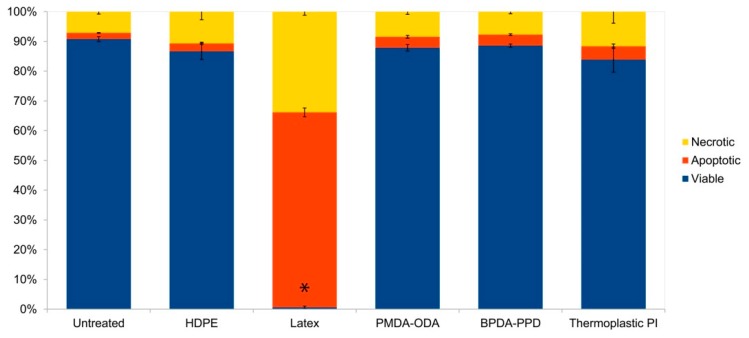
Cell viability of SV-HCEC human endothelial cells after 24 h exposure (Polyimide (PI) samples in cell culture media at 37 °C for 48 h, 1 cm^2^/mL media ratio) to extracts coming from various common PIs: PMDA-ODA (Kapton), BPDA-PPD (thermally imidized PI2611 precursor), and polyimidic adhesive (thermally imidized HD3007 precursor). Negative controls: untreated cells, high-density polyethylene (HDPE). Positive control: natural latex rubber. Reproduced from [30], with permission from © 2015 John Wiley and Sons.

One of the few in-depth studies treating the interaction between cells and commercial PI leachables investigates the effect of extractables coming from three commonly employed PIs (preimidized Kapton foil and in-house thermally imidized PI 2610 and HD 3007 precursors) on mitochondria and cell viability [30]. The research follows ISO 10993 protocols and is focused on some of the most expected leachables like PAA, incompletely cyclized products and residual solvents (in this case, N-methyl-2-pyrrolidone and gamma butyrolactone). The study does not cover degradation products since they are mostly expected after long-term in vivo employment. It was found that the incubation of human capillary and microvascular endothelial cells with extracts coming from the three PIs did not generate any significant stress or cytotoxicity, since no relevant mutations in the marker of mitochondrial stress (membrane potential) or the more severe markers of cell viability (apoptotic and necrotic cell death) were detected (Figure 4).

The success of this assay has a manifold explanation that mostly resides in the high-temperature (300 °C) gradual treatment which is used to imidize the films. On one side, the completely imidized PI chains are highly inert, while the unavoidable small portion of uncyclized backbones is reduced to a minimum. At the same time, the ineluctable residual solvent content, already regarded as of low toxicity, is too low to interfere with the final result. Moreover, the two psPIs are heavily cross-linked and therefore any macromolecular mobility or solvent and small components elution is very limited. Although promising, these results need to be followed by further research dealing with any potential cytotoxicity of aged PIs.

Another study follows the cytotoxicity of four noncommercial PIs films cast from N,N-dimethylacetamide and containing the same diamine and several commonly employed anhydride fragments (SPAB-xxDA, their chemical structure is shown at the end of Table 2) [55]. All of the investigated samples did not provide any leachables that could prove toxic for human dermal fibroblasts, as determined by indirect and direct (days 1, 3, and 6) contact assays. The latter was used to build an anhydride-dependent biocompatibility scale which, at this point, cannot be completely comprehended: 6FDA > BPDA > PMDA > ODPA. Nevertheless, the study concludes that the 6F (hexafluoroisopropylidine) structural motif limits the absorption of human fibroblast and contributes to improved biocompatibility. It would be really helpful to see if the biocompatibility differences between PIs or the above order would be kept when changing the starting amine. This work represents an interesting starting point for more detailed research focused on the role of various functional groups upon the biocompatible characteristics of a polyimidic material.

### 4.2. Surface

As for any given polymeric material [17,18], the wettability of a PI surface, commonly expressed by contact angle values, has a major influence on its biological response. Enhanced protein adsorption and cell adhesion are usually obtained in the case of more hydrophilic surfaces. However, as detailed previously, any increase in water uptake can have a negative impact on the long-term PIs’ mechanical features and reliance. Nevertheless, if, for example, a PI film is intended for cell or tissues culture usage, then it needs a surface of higher hydrophilicity, which, in the case of this type of polymers, can be attained by laser or plasma treatment [45,50,62,63] or chemical modifications [64,65,66,67,68]. The field of surface modifications of PI materials is so vast, that it would actually require a review of its own and therefore is not treated herein.

**Figure 5:**
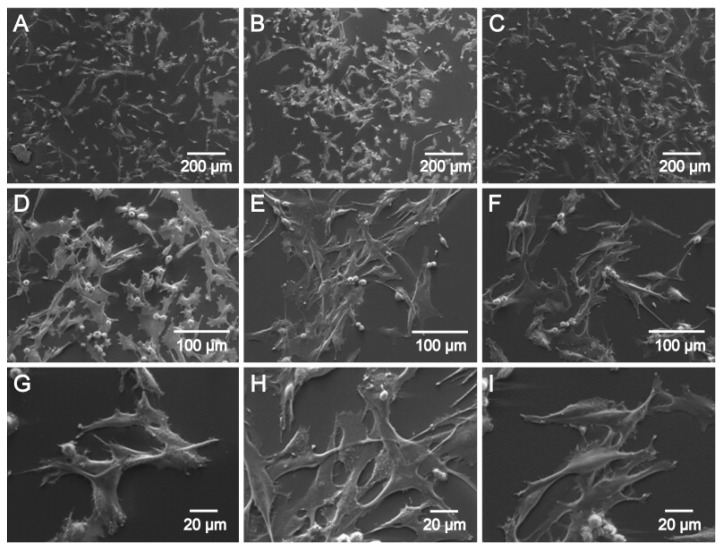
SEM micrographs (three different magnifications) showing the prevalence, morphology, and adherence of BHK-21 cells at 24 h after cultured on different polyimide surfaces: (**A**,**D**,**G**) conventional, non-photosensitive PI2525 cured at 200 °C; (**B**,**E**,**H**) photosensitive PI2771 cured at 200 °C; (**C**,**F**,**I**) photosensitive PI2771 cured at 350 °C. Adapted from [28], with permission from © 2010 Elsevier.

These observations were confirmed by cytotoxicity assays of commercial PIs. For example, the conventional PI2525 film has a higher hydrophilicity (roughly 25° smaller contact angles) and surface free energy (a surplus of ca 22 mJ/m^2^) as compared to the homologous photosensitive PI2771, which results in a small increase in the adhesion and proliferation of BHK-21 (baby hamster kidney cells) fibroblasts, as it can be observed in Figure 5.

The same rule of thumb (higher hydrophilicity for better compatibility) is available for PI biomaterials that come in contact with blood since surface energy and wettability drive the adsorption of plasma proteins and ulterior adhesion of platelets to their surface. Blood compatibility implies restricted, if any, platelet adhesion and activation, and thrombogenicity [18]. Even if the ultimate evaluation of hemocompatibility needs to be based on in vivo assays, in vitro investigations and even theoretical calculations can be valuable in this regard.

For example, the theoretical assessment of blood compatibility can be done by computing the spreading work of some basic blood cells and proteins on the surface of the polymeric material. This was applied to two PI materials: a noncommercial film containing alicyclic sequences, EPICLON-PPD (for its structure, see Table 2) and the conventional, aromatic Kapton film [20]. The theoretical study judiciously estimated a stronger non-adsorbent (of blood components) character and a subsequently more hemocompatible behavior for the aliphatic material as compared to the aromatic one. Even if further experimental confirmation of these results is needed, they are already sustained by the higher contact angle and lower polarizability values of the noncommercial film, which, according to the aforementioned observations, support the theoretical outcome. Other studies confirmed experimentally that various commercial PIs are hemocompatible, also underlying certain variability across formulations [30].

**Figure 6:**
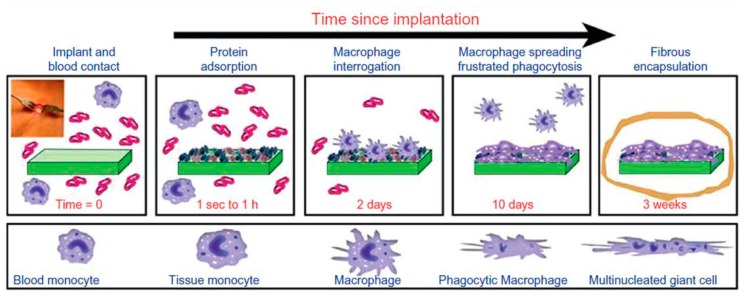
The in vivo composite reaction to synthetic biomaterials: timeline and monocyte/macrophage phenotypes. Adapted from [17], with permission from © 2015 Elsevier.

The discussion about surface biocompatibility needs more detail since the main bio-application of PI materials envisages implanting, and therefore they need to show a minimal effect on the body. Any implantation of a biomaterial (which has already been certified as leachables-free and overall non-toxic by standardized in vitro assays) triggers a mild physiological inflammatory response. This reaction starts with immediate protein adsorption and evolves after roughly three weeks with macrophages and giant cells walling the implant with a fibrous capsule (Figure 6) [17].

This aggregate, chemical, physical, and biological process is summed up as the foreign-body response (FBR) and depends on many factors, from leachables and endotoxins to size and mechanical features of the implanted material or its biospecific interactions with its surroundings. The ultimate “biocompatible” attestation of a biomaterial is given by an unsophisticated FBR resulting in a stable, nonadherent, well-defined fibrotic tissue enclosing the implant. Of course, this capsule should not harm the implant in any way, and it could actually prove a valuable asset, by supplying additional spatial fixation. Several studies regarding the implantation (mostly focused on the peripheral and central nervous system) of various PI-encapsulated devices evidenced that this type of material exerts a mild, quiescent FBR (confined to the immediate vicinity of the material) and qualifies as biocompatible [16,25,35,36,37,38,39,42,43].

### 4.3. Mechanical Effects

As indicated earlier, there are several mechanically-driven interactions between the implant and its environment which affect the success of an implant, and they can be summed under the term structural biocompatibility [25]. Any mechanical mismatch between the hard biomaterial and the soft target tissue in terms of flexibility/rigidity, shape (sometimes even texture) or weight leads to micromovements, cell, tissue, or material irritation and damage, and, in the end, to implant failure. There are three types of factors responsible for such a drawback. First, there is the implant manufacturer, which needs to make sure that the implant is not rubbing or irritating the tissue by, e.g., replacing a sharp-edged design with a more rounded one. Second, there is the surgeon performing the operation, which needs to minimize such a rubbing-inflicted irritation by proper placement and anchoring [17]. Still, most of the responsibility for mechanical mismatch belongs to the polymeric material, through its mechanical and biological functionalities.

Polyimidic materials are the prime polymeric materials of many implant-related applications (especially the demanding neural ones) since they provide a wide range of mechanical features, from easy flexibility to straight stiffness, and enable the facile construction of multilayered implantable devices. However, they are still prone to mechanical mismatch, by means of manufacturing, incompatible mechanical features, and deterioration induced by the biological media.

**Figure 7:**
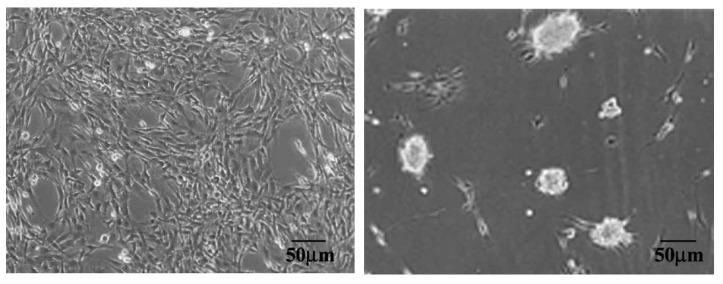
Phase-contrast images of fibroblast cells at 48 h after cultured on unrubbed (left) and rubbed (right) surface of a synthetic fluorinated polyimide (6FDA-6FAP). Adapted from [53], with permission from © 2002 Elsevier.

For instance, mechanically-inflicted surface modification can alter the interaction of a PI material with living cells and plasma proteins. The rubbed surface of a noncommercial fluorinated PI (6FDA-6FAP, Table 2) displayed dissimilar hydrophobicity and roughness as compared to the starting material, which resulted in different biological behavior in terms of plasma protein adsorption, platelet adhesion, and fibroblast proliferation [52]. Rubbing proved an advantage in this particular case since the generated nano-ordered domains determined selective protein adsorption and spheroidal aggregation of cells (Figure 7). The key point of the study is that this type of mechanical modification has a decisive impact on the proper functioning of an implant.

In another case, the inherent mechanical characteristics of the PI material do not fully comply with its in vivo intended use. For example, the photosensitive Probimide 7520 was tested for intracortical neural implants due to its proven biocompatibility and flexibility. The latter mitigates tissue irritation coming from the micromotion of a hard PI material on the brain’s soft tissue [69]. However, this flexibility can be a disadvantage: the PI-based electrode bends and overarches during implantation and, as a consequence, it cannot pass the brain’s pia mater. The structural stiffness can be resolved, for example, by using an extra layer of silicon, but this will complicate manufacturing, reinitiate biocompatibility screening and add a new source of delamination or degradation [49].

In some cases, this behavior is just a case of a mechanical mismatch between the implanted PI and the target tissue, which could be ruled out by selecting another PI material. As a counterexample to the above, another flexible psPI, namely Durimide 7510, did not induce any negative mechanical effect after 6 months of epiretinal implantation in rabbit eyes [41]. In this case, the flexibility of the PI (also doubled by a round-edged design) helped to eliminate any mechanical compression on the retina and allowed relatively simple and convenient implantation, as it will be detailed in the section dedicated to in vivo experiments.

### 4.4. Manufacturing

The construction process of any given implant, even in the early production stages of raw materials can also inflict a different biocompatible behavior. In the case of PI films, the most important preparation step is the imidization of the material. This is usually performed by curing the film at temperatures ranging between 150 and 400 °C, depending on the structure or on the manufacturers’ indications for the commercial materials [1,58]. The cyclization temperature can have a major influence on the adhesion, proliferation, and morphology of certain cells and, by extension, on the FBR behavior, since PI reach their top chemical stability only if completely cured (the maximum possible cyclization eliminates or minimizes PAA residues and solvent traces).

**Figure 8:**
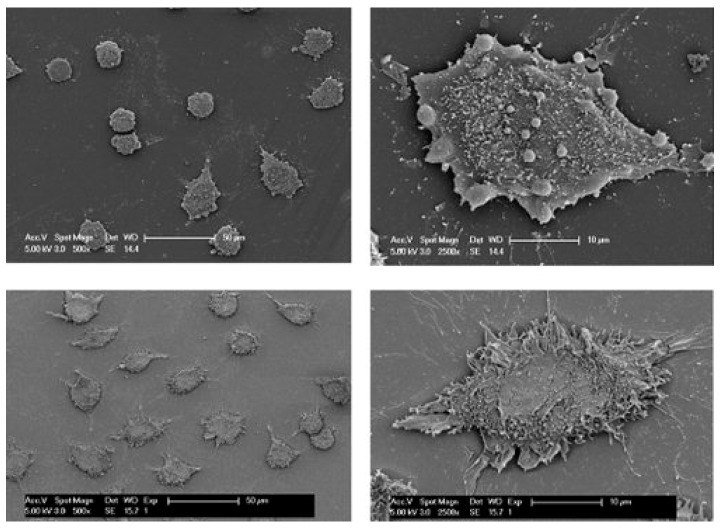
Scanning electron microscopy images (500×, 2500×) showing the viability and adherence of L929 fibroblast cells at 24 h after cultured on the surfaces of conventional PI2611 cured at 200 °C (top) and at 400 °C (bottom). Reproduced from [27], with permission from © 2008 John Wiley and Sons.

One experimental argument of this hypothesis was provided by the treatment of spin-coated conventional PI2611 precursor at two different temperatures, followed by autoclave sterilization and fibroblast populating experiments. The film cured at 200 °C showed a weak cellular adhesion and proliferation, while its counterpart cured at 400 °C displayed superior fibroblast adhesion and viability (Figure 8) coming from a more efficient elimination of chemical residues and/or solvent traces [27].

The same conclusion was drawn by curing a commercial psPI, PI2711, at two different temperatures, 200 °C (Figure 5B,E,H) and 350 °C (Figure 5C,F,I), respectively, and observing some variations in the adhesion and proliferation of BHK-21 fibroblasts, along small changes in the surface free energy and zeta potential (and surprisingly constant contact angle values).

**Figure 9:**
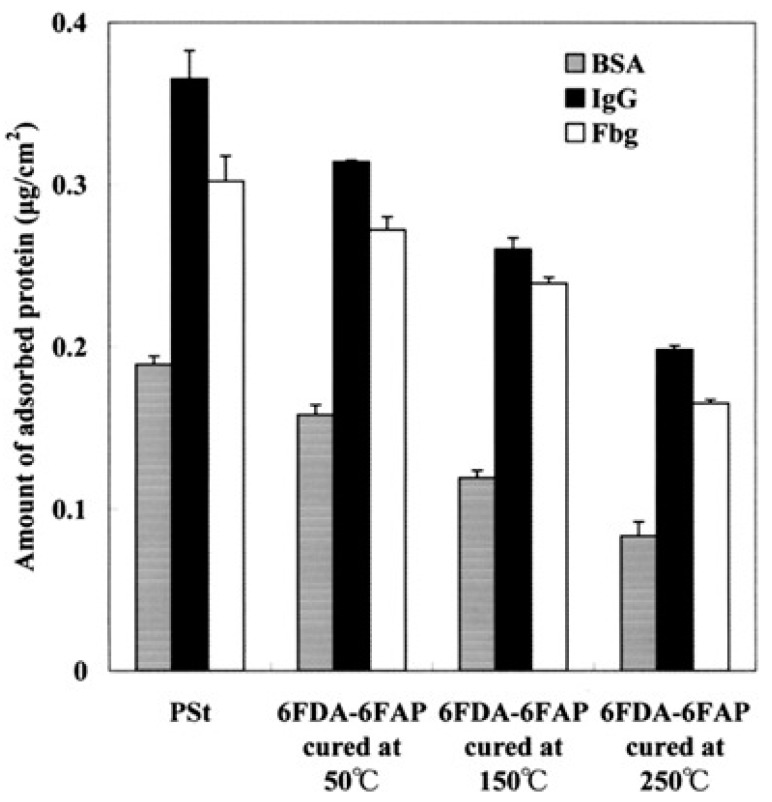
The amount of three adsorbed proteins on the surfaces of the same 6FDA-6FAP fluorinated polyimide membrane cured at three different temperatures (pH 7.4; polystyrene (PSt) control). Reproduced from [53], with permission from © 2002 John Wiley and Sons.

The straightforward dependence between imidization temperatures and biological behavior was also observed in the case of a fluorinated PI (6FDA-6FAP; Figure 9). In this particular instance, protein adsorption, neutrophil adhesion, and complement activation are inversely proportional to temperature, showing better values at lower cyclization temperatures. This behavior is determined by a high temperature-induced rearrangement of molecular fragments at the outermost surface, which results in low surface energy and high hydrophobicity [53].

Further studies employing other PI materials are needed in order to fully grasp the effect of imidization temperature on their micro- and macro-molecular structure, surface, hydrophobicity, and subsequent biocompatible behavior.

From another point of view, it must be underlined that many of the results discussed in the last two subsections were obtained in static conditions. The broad range of flow conditions implied by tissue- and blood-contact devices brings additional variables and unknowns to the issue and protein adsorption, cellular adhesion, or complement activation may present a significant variation from the static experiments.

Further, the manufacturing stages of implantable bio-MEMS, for example, need to reduce any source of leachables and minimize their toxicology. Once again, PIs are quite resistant to micromachining techniques, so the toxicity risk in this regard is minor but still noteworthy. However, these well-known general features of PIs do not exclude the in-depth physicochemical characterization of any given PI-based material and of its final products (a biocompatible PI does not guarantee a biocompatible PI-based device). Special attention must be dedicated to any modification in the composition and properties of the PI material which could arise from hydrolytic or enzymatic degradation during its in vivo employment, especially since it is the case of devices conceived for long-term implantation.

## 5. Biocompatibility Screening of Polyimides by In Vitro Methods

Cytotoxicity evaluations represent the first stage of the biocompatibility assessment of any given biomaterial and they enable qualitative and quantitative evaluations of the possible biological risk of their usage. Despite their specificity shortage, these tests represent the most suitable, inexpensive, reliable and, very important, reproducible means to review the biological response induced at the cellular level and to simulate, with some limitations, in vivo circumstances.

Besides the available standards and regulations, the selection of cells and cellular models employed in these studies is (or should be) determined by the envisaged outcome. Usually, the basic cytotoxicity screening is performed on common cultures of isolated, immortalized cells (like L929, HeLa, 3T3). The more thorough cytocompatibility investigations use specific cell lines (like animal or human endothelial, epithelial cells), according to the foreseen biological interactions and medical usage. Combined assays of both types, either in direct or indirect contact mode, can deliver a more accurate image of a material’s cytotoxicity and should be applied to complement its physicochemical evaluation.

**Figure 10:**
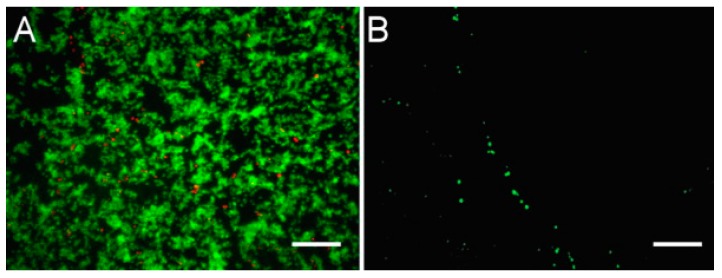
Cell viability assay examination of BHK-21 fibroblast cells at 24 h post-culture on PI2525 polyimide: (**A**) live (viable: green color)/dead (non-viable: red color) staining of cells on the surface of PI2525; (**B**) positive control (latex rubber). Scale bar: 200 µm. Reproduced from [28], with permission from © 2010 Elsevier.

Even if PIs are not yet certified by ISO standards, there are several research papers in which their low, if any, cytotoxicity is demonstrated. Some of the most solid ones are being cited in this section, while an example of a successful cell viability assay is presented in Figure 10. However, the available scientific literature on the topic provides a wide range of methods and cell lines, and an explicit definition of assessment conditions is needed in order to perform any reliable interpretation or comparison of the assay’s results [17,18,57]. For instance, the first systematic in vitro evaluation of commercial polyimidic products dates back to 1993 and does not follow the currently available ISO guidelines, but another set of prior, nevertheless trustworthy standards [15]. Even if this is deservedly one of the most cited papers when PIs are described as “biocompatible”, any judgment based on it or comparison with the current guidelines is not reasonable.

Various commercial and noncommercial PI materials were proven as non-toxic by a multitude of in vitro, direct, or indirect, cytotoxicity assays. Most of them specifically declares to comply with the ISO guidelines, and investigate the viability, proliferation, degeneration, and lysis of different common cell types:

- L929 mouse fibroblast cells (Kapton, PI2611, Durimide 7020, Durimide 7510, PI2525, 6FDA-6FAP, EPICOLN-PPD) [27,41,44,48,51,52],
- 3T3 mouse fibroblasts cells (Probimide 7520) [49],
- OP9 mouse stromal cells (Neopulim) [19],
- rat primary Schawnn cells (Kapton, PI2611, Durimide 7020) [27],
- rat astroglial cells (unknown PI trade name) [70],
- bovine endothelial cells (unknown PI trade name) [70],
- BHK-21 baby hamster kidney fibroblasts (PI2525, PI2771) [28],

or more specific cell lines:

- human retinal pigment cells (PI2525) [47],
- AGSE human gastric adenocarcinoma cells (unknown PI trade name) [70],
- human mesenchymal stem cells (Kapton) [20],
- HeLa, human epithelial cells (6FDA-6FAP) [54],
- SV-HCEC human endothelial cells (Kapton, PI2610, HD 3007) [30],
- human dermal fibroblasts (SPAB-xxDA) [55].

**Figure 11:**
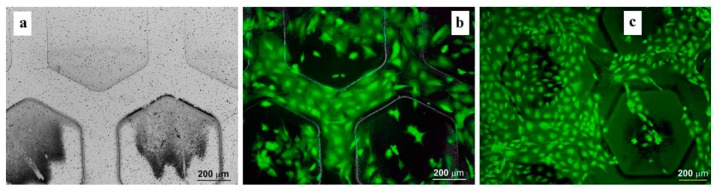
Optical micrographs of two cell lines on the surface of a polyimide sample with carbon patterns: (**a**) carbon layer pattern; (**b**) rat astroglial cells; (**c**) bovine endothelial cells. Reproduced from [70], with permission from © 2008 Springer Nature.

The low cytotoxic outcome of these tests and the well-established PIs’ surface treatment methods were further combined to attain an interesting controlled positioning of living cells along within a given pattern [70]. A KrF laser-based technique was employed to garnish the surface of a commercial psPI with an extra carbon layer and to manipulate its interaction with various cell lines (Figure 11). For example, bovine endothelial cells proved quite immune to this surface modification, while rat astroglial cell lines showed guided adherence and proliferation by avoiding the nonadherent carbon layer, an observation which can prove very helpful for further cellular culture-related applications.

## 6. Biocompatibility Screening of Polyimides by In Vivo Methods

In vivo screening is the next level of biocompatibility testing, the critical step in the evaluation of the biological response generated by a material. While well developed and comprehensive, the in vitro methodology is not able to accurately model many of the aspects implied by a clinical biological reaction. The latter brings additional hurdles like high controversy, ethical issues, premium costs, cumbersome bureaucratic challenges, etc. [57].

Several commercial PIs have been the subject of in-depth in vivo studies that authenticated their compatibility with living biological organisms. Most of the reachable clinical trials involved defined polyimide structures listed in Table 1 and Table 2 (Kapton, Durimide 7050, Durimide 7510, PI2611, PI2525, U-Varnish S) but also a few unknown commercial PIs which are simply and frustratingly named “polyimides”. This review is not intended to reproduce or castigate the conclusions of these articles, but to underline some of the most spectacular and meaningful cases of PI’s in vivo testing as the ultimate and undeniable proof of their biocompatibility.

**Figure 12:**
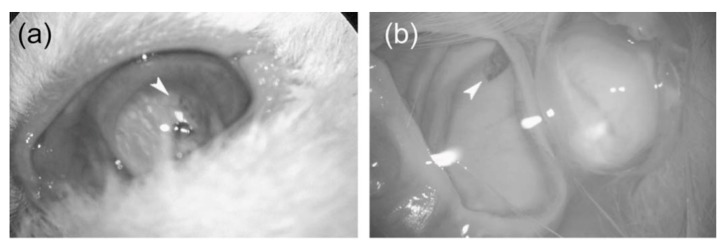
In vivo biocompatibility tests performed on a polyimide (PI2525) microelectrode array (3 × 3 mm, 18 µm thick polyimide support) for retinal stimulation: (**a**) fixed polyimide-based array at 12 weeks after implantation in rabbit eyes; (**b**) free-floating polyimide-based array at 12 weeks after implantation in rabbit eyes. Adapted from [47], with permission from © 2004 Elsevier.

The first example in this regard deals with a flexible commercial cPI, PI252, which was used to build the components of an artificial retina with the aim to stimulate retinal neurons in cases of degenerated photoreceptors [47]. Its in vivo biocompatibility was tested at 12 weeks post-implantation in rabbit eyes, as a free-floating and a fixed implant (Figure 12). Both of them proved good biological and mechanical stability and no inflammatory response, the electroretinogram and microscopic investigations not being able to find any disparities between the healthy and the transplanted eye.

**Figure 13:**
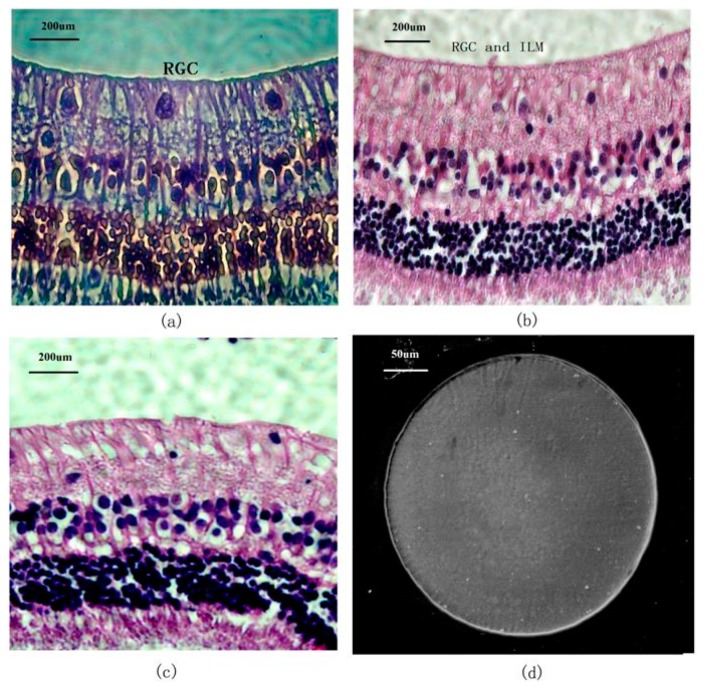
Long term in vivo biocompatibility evaluation of a microelectrode array based on photosensitive Durimide 7510 polyimide layer implanted within a rabbit eye: (**a**) phase-contrast image (400×) of the inner retinal morphology at 6 weeks post-implantation (RGC: retinal ganglion cell layer; ILM: inner limiting membrane); (**b**) light microscope image (400×) of the inner retinal morphology at 6 months post-implantation; (**c**) light microscope image (400×) of the control rabbit retina; (**d**) SEM micrograph of the undamaged implanted electrode at 6 months post-operation. Reproduced from [41], with permission from © 2013 BioMed Central, under CC BY 2.0.

As a complement of the former, the second example of successful PI implantation is related to a commercial psPI (Durimide 7510) used in the preparation of MEMS for epiretinal electrical stimulation. Its reliability and stability were assessed by chronic 6-month implantation in rabbit eyes, which showed a nonirritant, biologically safe PI-based device with high biocompatibility and no mechanical pressure or mismatch. The study details the lack of cytotoxicity of the thin PI sheet, which did not bring any injury or modifications to the inner retina or its layers, as it can be observed in Figure 13a–c. Moreover, the PI material was immune to sterilization and the harsh biological environment, and, also important, no delamination was noticed 6 months after the implantation (Figure 13d) [41].

**Figure 14:**
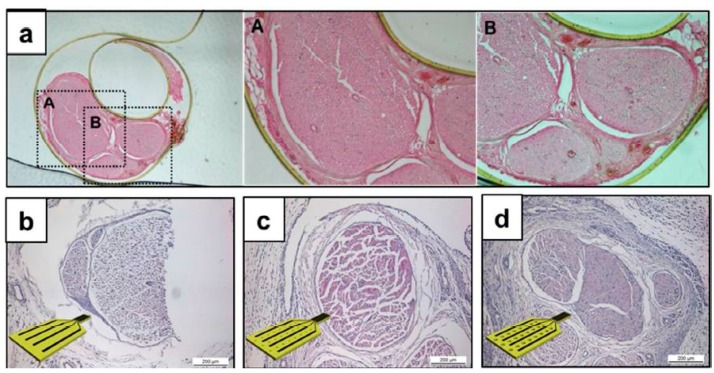
Histological images of nanofiber, polyimide (PI1338)-based electrodes after implantation in the sciatic nerve of a rat: (**a**) pristine electrode straight after implantation (**A**,**B**) magnifications of the contact area between tissue and electrode); (**b**) pristine electrode at 4 days post-implantation; (**c**) electrode with small holes at 4 days post-implantation; (**d**) electrode with large holes at 4 days post-implantation. Reproduced from [36], with permission from © 2017 American Chemical Society.

The mechanical mismatch issue encountered in neuroprosthetics may find a solution by using electrospinned PI nanofibers instead of casted PI films. A neural electrode based on such highly flexible and permeable polyimidic nanofibers provides a smaller contact area and reduced neural compression (Figure 14). In exchange, these features enable a decrease in the shrinkage of the nerve tissue and long-term (12 weeks), steady recordings of neural signals [36]. Moreover, biologically active principles can be loaded on such nanofibers to enhance its affinity with the sciatic nerve tissue or to pave the way for very interesting neural drug-delivery applications.

At this point, we must note that the same cPI material steps forward from the handful of in vivo tested polyimidic materials: the BPDA-PPD macromolecular architecture. Most likely known by the trade names of its liquid precursors, PI2611 and U-Varnish-S, this film was heavily tested as a passive component in a broad range of applications. Its uses span from retina stimulation and blood sensing to cortical recordings, phantom pain relief, and intra-neural stimulating electrodes for the peripheral and central nervous systems [25].

**Figure 15:**
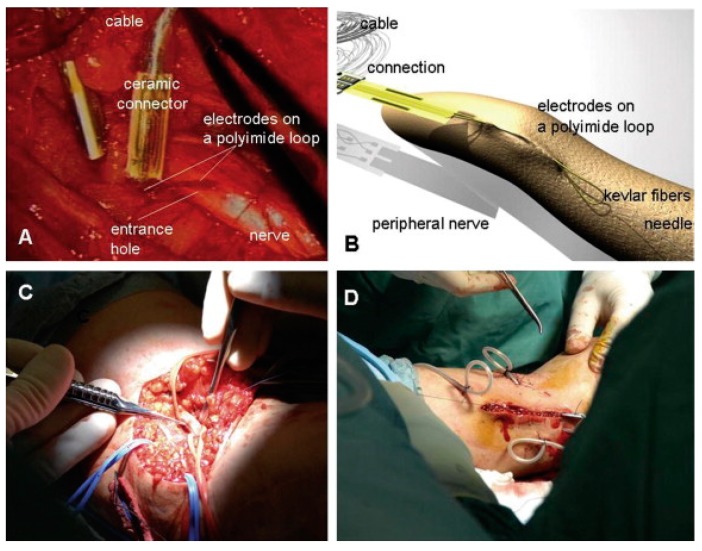
Surgical implantation of a tf-LIFE4 intraneural electrode implant based on a polyimide (PI2611) substrate (10 µm in thickness and 5 cm in length). (**A**) A magnified photographic view of implant components during surgical implantation; (**B**) schematic illustration of the overall tf-LIFE4 implant system; (**C**) photographic view median nerve, tf-LIFE4 electrode and cable during surgical implantation; (**D**) external transit cables after implantation. Reproduced from [33], with permission from © 2010 Elsevier.

Perhaps the most tremendous example of in vivo biocompatibility of a PI-based material comes from an intraneural flexible electrode built on this commercial PI, tf-LIFE4, which was implanted in a human amputee with the aim of controlling a robotic hand (Figure 15) [33]. After a 4-week in vivo test period (as imposed by European Authorities), the implant proved biocompatible and stable even when the patient was performing everyday life activities. However, a fibrotic tissue reaction was detected by visual inspection during the removal of the device, but ethical reasons did not allow its histological confirmation. The device itself and the cited study signify a real breakthrough in the field of implantable robotics for amputees and also open great opportunities for polyimidic materials.

There are also other available examples of intraneural flexible electrodes based on PI materials, with TIME and SELINE flexible electrodes being the main competitors of tf-LIFE. The main difference between the three is the direction in which they are implanted and the three-dimensional geometry of the device. In all cases, the PI2611 polyimide is the material of choice for insulation and mechanical support attainable through standard photolithographic techniques. The available studies concur that this polyimidic material provides a good compromise between biocompatibility, mechanical features, and insulating efficacy [27,39,46]. All these electrodes showed promising results in functionality, selectivity, invasiveness, and biocompatibility studies performed on animal models and human volunteers. The main application limits are related to the overall device design, mechanical anchorage to the nerve, and post-implantation stability. Further research is needed to improve the stability of the electrodes for acute and chronic studies and to comparative asses the devices’ chronic functionality. This type of study will also contribute greatly to the currently reduced body of knowledge related to the long-term stability of polyimides in vivo conditions.

## 7. Conclusions and Future Directions

Biocompatibility assessment of a polyimidic material designed for the medical field must comply with a composite enterprise of many variables, from the chemical nature or physical characteristics of the envisaged PI to the tissue contact span or the nature of that given tissue. A judicious screening order should start with an in-depth evaluation of all available data, followed by the integration of chemical and physical investigations, basic and specific in vitro screening, and legitimate in vivo characterization.

Biomedical applications are more demanding than ever for novel, biocompatible, skillful materials which can simultaneously provide lightweight, superior mechanical features and uncomplicated manufacturing. PIs resolve many of these requirements by an unparalleled combination of inherent characteristics, ease of tuning, facile production, and manifold utility. Several commercial and noncommercial materials developed from polyimidic building blocks have been severely tested by both in vitro and in vivo methods in the last two decades which lead to a quite impressive, yet mostly disconnected body of knowledge. Even if PIs are not yet certified by ISO standards, a respectable amount of research authenticate their non-toxic nature in vitro and demonstrate cell viability and proliferation of different common cell types on various PI materials. Moreover, a small number of commercially-available PIs were labeled as “biocompatible” by in-depth, acute, and chronical in vivo studies on animal models and, in few cases, even on human volunteers.

The positive results provided by these scrupulous investigations enable a wide exploration of polyimides in various biomedical applications, from micropatterned cell substrates and blood sensing to cortical recordings, retina stimulation, and intra-neural stimulating electrodes for the peripheral and central nervous systems.

It is safe to reason that commercial or synthetic PI materials are far from reaching their peak usage in medical devices and implants. On one side, many studies are dedicated to providing tailored PIs specially designed for the biomedical area since the demand for unique implants can be also met through polyimidic materials. Moreover, recent studies report physically or chemically modified and composite PIs for the same type of applications. On the other hand, intense research is focused on improving the functionality and stability of medical devices based on the “classic”, commercial PIs described herein. Despite all this, it is still difficult to discern which PI-based material works best for different biomedical usages.

An outlook into the future of PIs in the biomedical area can follow multiple directions. First, more studies are needed to access a straight line between the chemical blueprint of a polyimidic material and its biocompatibility. The data presented here come close to a basis for tailoring PIs to achieve in vitro biocompatibility. Even so, correlations between important factors like curing temperature, interfacial free energy, or molecular weight and biocompatibility are still unreachable. Additionally, further research dealing with any potential cytotoxicity of aged PIs is needed.

Second, PI-based medical devices are far from clinical controlled performances. Further in vivo assays of recognized or new PI materials and their outcomes’ in-depth comparisons could be a first step in solving this issue. The in vivo studies should also address the long-term chemical and biological inertness of PIs as the main prerequisite of medical devices’ biocompatibility. There is a lack of data related to changes in the chemical compositions, mass loss, and macroscopic behavior of PIs after long-term in vivo employment. At the same time, the in-depth analysis of specific tissue responses and FBRs triggered by PIs is required. These missing pieces in the body of knowledge related to PIs are a first limit to their expansion in the biomedical market. A rule of thumb for any of these biocompatibility assays should be the use of validated and standardized test methods. Otherwise, any applicable, comparable, and conclusive results would be out of reach.

In another direction, polyimidic materials alone or intraneural electrodes based on them are still prone to mechanical mismatch, a defect which hampers the development of neuroprosthetics. While the latter is a matter of device design, new PIs must be tested to mitigate the material-related difficulties. Another issue to be addressed in this area is the water uptake of some PIs, especially of the psPI type. Moisture adsorption causes stretching and has a plasticizing effect which limits their application. Finally, the delamination of metals from PIs remains a topic of concern for the long-term durability of bio-MEMS.

The main drive of research in the field of polyimides will logically remain focused on novel, polyimidic materials of superior performance. However, special interest must be directed towards their ability to make it even further in the biomedical area, with proper (especially in vivo) biocompatibility being the first item on a long checklist. The ability of polyimides to tolerate or even adapt to the circumstances created by a living environment is the pivotal element in the production of stable, reliable medical devices destined to long-term, nontoxic usage. A couple of success stories cited herein pave the way for more intensive research focused on the biocompatibility of old or new polyimides as to further improve their bio-performance and safety and to unlock unmet biomedical possibilities and applications. Nevertheless, an undeniable change has occurred in the way polyimides are regarded by the biomedical field and the concepts of “biocompatibility” and “polyimides” are more congruent than ever.

## Figures and Tables

**Table 1:** Structure, trade name, and producer of commercial polyimides (or precursors) tested for their biocompatibility.

Structure of Polyimide (or Precursor ^1^)	Trade Name ^2^; Producer Biocompatibility Studies [References]
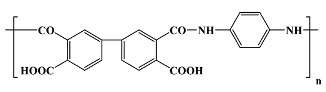	PI2611 ^1^; Du Pont in vitro; in vivo [7,16,27,29,30,31,32,33,34,35,36,37,38,39,40]
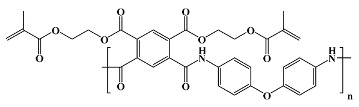	Durimide 7000 ^1^; Fujifilm in vitro; in vivo [27,29,36,41,42,43]
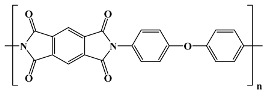	Kapton; Du Pont theoretical; in vitro; in vivo [15,20,27,30,44,45,46]
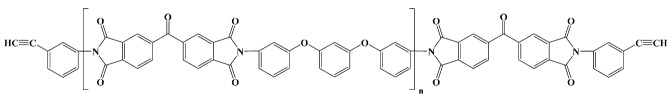	Thermid EL 5010; National Starch and Chemical Co in vitro [15]
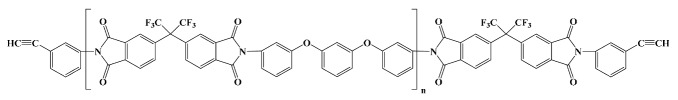	Thermid EL 5512; National Starch and Chemical Co in vitro [15]
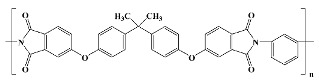	Ultem 1010; GE Plastics in vitro [15]
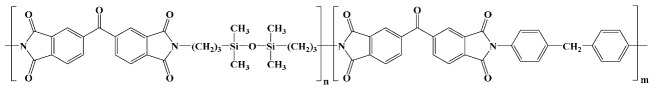	Silbem; GE Plastics in vitro [15]
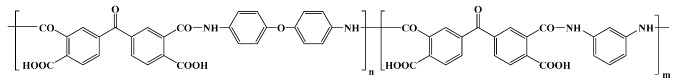	PI2525 ^1^; Du Pont in vitro; in vivo [28,47,48]
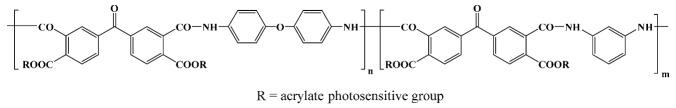	Pyralin PI 2700 ^1^; Du Pont in vitro [28]
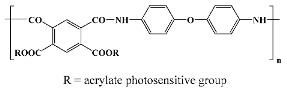	Probimide 7500 ^1^; Fujifilm in vitro; in vivo [49]
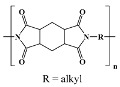	Neopulim; Mitsubishi in vitro [19]

^1^ structure is represented as the commercial polyimide precursor, but all biocompatibility tests were performed on the imidized form of the polymeric material; ^2^ common tradenames or abbreviations for the monomers: PMDA (pyromellitic dianhydride), BPDA (biphenyl-tetracarboxylic acid dianhydride), PPD (*p*-Phenylenediamine), 6FDA (4,4′-(hexafluoroisopropylidene)diphthalic anhydride), ODPA (4,4′-oxydiphthalic anhydride), ODA (4,4′-oxydianiline).

**Table 2:** Structure and abbreviation of synthetic polyimides (or precursors) tested for their biocompatibility.

Structure of Polyimide (or Precursor ^1^)	Abbreviation Biocompatibility Studies [References]
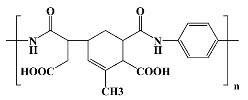	DOCDA-PPD ^1^ theoretical [50]
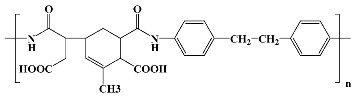	DOCDA-DDE ^1^ theoretical [50]
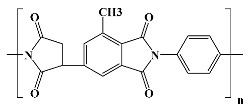	EPICOLN-PPD theoretical; in vitro [20,51]
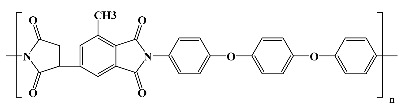	EPICOLN-APB theoretical; in vitro [51]
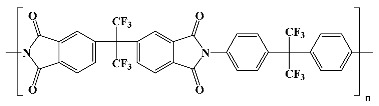	6FDA-6FAP in vitro [52,53,54]
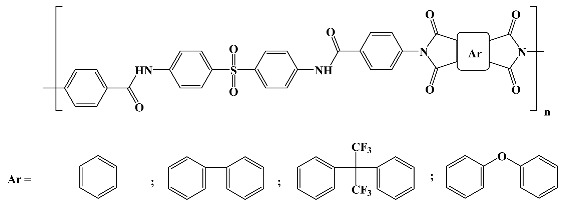	SPAB-xxDA in vitro [55]

^1^ biocompatibility tests were performed on the polymeric precursor (polyamidic acid); ^2^ common tradenames or abbreviations for the monomers: DOCDA (5-(2,5-dioxotetrahydrofuryl)-3-methyl-3-cyclohexene-1,2- dicarboxylic anhydride), DDE (4,4′-diaminodiphenyl ether), APB (1,3-bis(3-aminophenoxy) benzene), 6FAP (2,2-bis(3-amino-4-hydroxyphenyl) hexafluoropropane).
